# Supplementary material for: A SNP-based capture and clustering workflow to assess donor-derived cell-free DNA in transplantation
Source: PLoS One. 2026 Feb 2;21(2):e0342082. doi: 10.1371/journal.pone.0342082 (PMC12863547; doi:10.1371/journal.pone.0342082)
Supplement: Attachment — Submitted filename: Response to Reviewer 2.docx [file pone.0342082.s004.docx]

**Reviewer 2:**

We sincerely thank Reviewer 2 for your constructive comments and insightful suggestions. We have revised the manuscript accordingly to improve clarity, rigor, and clinical relevance. Below, we provide a detailed point-by-point response.

1. Clinical Correlation and Utility

The article lacks direct correlation analysis with clinical outcomes (e.g., biopsy-proven rejection, renal impairment). Given that data from over 650 samples are available, it is recommended that the authors include a preliminary analysis—such as a graph showing the dynamic changes in dd-cfDNA (as detected by this method) in one or two cases during rejection events. This would significantly enhance the article’s persuasiveness and clinical relevance.

Additionally, while Table 2 demonstrates concordance between clustering-based estimates and direct genotypic calculations, the manuscript does not compare the sensitivity and specificity of the clustering method versus traditional methods in diagnosing rejection episodes. Including such a comparison would better highlight the clinical utility of the approach.

[Response]

We appreciate the reviewer’s suggestion. To illustrate the clinical relevance of our method, we have added a representative case in the Supplementary Information (Supplementary Figure S4 Fig.) showing longitudinal dd-cfDNA dynamics around a biopsy-proven rejection event. In this example, dd-cfDNA increased in close temporal association with the rejection episode, and the elevation was consistently detected both with and without the use of pre-transplant reference data. This addition provides a concrete case-level demonstration of the method’s utility.

Our study was primarily designed to establish analytical validity and concordance with direct genotyping, as summarized in Table 2. While Table 2 demonstrates strong agreement between clustering-based estimates and direct calculations, the dataset was not structured to allow a robust ROC analysis of sensitivity and specificity against biopsy outcomes. Such analyses are ongoing within organ-specific cohorts and will be reported separately by participating institutions. We have revised the Discussion to clarify this point and to reference the new Supplementary Figure (S4 Fig).

2. Cost and Efficiency
The introduction mentions that existing NGS-based methods for estimating dd-cfDNA ratios are time-consuming. While the proposed clustering method appears to reduce analysis time, the manuscript does not discuss cost advantages over traditional approaches. Could the authors provide a comparative analysis of the costs involved in their method versus conventional techniques?

[Response]

We appreciate the reviewer’s constructive suggestion. In response, we have expanded the Discussion to clarify the relative cost and efficiency aspects of our approach. In general, dd-cfDNA quantification by NGS involves cfDNA purification, enrichment of informative SNPs, sequencing, and bioinformatics analysis. Our clustering-based strategy streamlines the analytical phase by allowing direct estimation from SNP counts without requiring donor genotypes, thereby reducing computational burden. Regarding enrichment methods, capture-hybridization typically requires >4 hours, whereas multiplex PCR may be faster, although overnight hybridization is often feasible without extending turnaround time in practice. In terms of reagent cost, the difference between capture-hybridization for up to 96 samples and multiplex PCR targeting several hundred SNPs is expected to be limited. These clarifications have been added to the Discussion section to better position our method relative to conventional approaches.

3. Population Specificity and Generalizability
The 300 SNPs selected were based on their frequencies in the Japanese population. It is unclear whether these polymorphisms are equally effective in other ethnic groups (e.g., Caucasians, Africans). The generalizability of the method and any adjustments that may be needed for its application in different populations should be discussed.

[Response]

We thank the reviewer for this important comment. The 300 SNPs used in this study are listed in Supplementary Table 1 and were selected as a subset of the 1,000 SNPs described in Reference 5. These loci were chosen because their minor allele frequencies (MAFs) are high (typically 0.4–0.5) in the Japanese population. Importantly, many of these SNPs also show comparable allele frequencies in European and African populations, supporting their potential applicability beyond the Japanese cohort. In practice, if certain SNPs prove to have low MAF in a given population, those loci can be easily substituted with alternatives of higher frequency identified from public population databases. Thus, while the present panel was optimized for the Japanese population, the overall framework is flexible and can be readily adapted to other populations. The generalizability of the method therefore derives from the clustering approach itself, rather than from a fixed SNP set.

4. SNP Panel Design and Sequencing Parameters
As discussed in recent literature [1], several factors critically impact dd-cfDNA assay performance:

Nucleosome Footprinting: The authors should clarify whether their 300-SNP panel avoids regions with strong nucleosome positioning, as differential nucleosome occupancy between donor and recipient tissues can lead to significant quantification bias.

SNP Count and Sequencing Depth: The choice of 300 SNPs and the achieved sequencing depth are not thoroughly justified. The authors should reference theoretical calculations to demonstrate that their panel size and depth are sufficient to reliably detect low-frequency heterologous signals (e.g., <0.5%) with acceptable confidence intervals. A discussion on whether this number provides a stable proportion of homozygous and heterozygous informative SNPs in their target population is warranted.

[Response]

We acknowledge that our 300-SNP panel was not explicitly designed to avoid genomic regions with strong nucleosome positioning. As cfDNA is known to exhibit fragmentation biases related to nucleosome occupancy, this may in theory introduce some degree of quantification bias. However, the selected SNPs are broadly distributed across the genome, and in our empirical dataset we did not observe clear evidence of systematic bias attributable to nucleosome positioning. We agree that future iterations of the panel could incorporate information from published nucleosome maps (e.g., ATAC-seq or MNase-seq data) to further minimize this potential confounder.

We thank the reviewer for raising this important point. As suggested, we have now included a theoretical justification of panel size and sequencing depth. Based on a binomial variance model, the variance of the observed ALT allele ratio for a single SNP is approximated by p(1–p)/R, where p is the true allele fraction and R is the read depth. When averaging across N_eff independent SNPs, the standard error becomes SE ≈ √{p(1–p)/(R·N_eff)}. For example, with R=1000 reads per SNP, N_eff=150 informative SNPs, and p=0.005 (0.5%), the SE is ~0.001, corresponding to a 95% CI width of ~0.2%, which is sufficient to distinguish signals below 0.5%.

Importantly, in our dataset the actual sequencing depth averaged ~10,000 reads per SNP (R1+R2 combined), substantially higher than the illustrative assumption. This further reduces the variance term and provides greater confidence in the ability of our 300-SNP panel to detect low-level dd-cfDNA with acceptable precision. A description of this rationale has been added to the Discussion section.

5. Low-Level Detection and Accuracy
In post-transplant monitoring—particularly after lung transplantation—dd-cfDNA levels are typically below 1% in the absence of rejection. As shown in Table 1, the measured values at mixing rates of 0.5% and 1% deviate noticeably from the actual values. I recommend increasing the density of sample testing within the 0%–1% mixing range to validate the method's reliability for low-level dd-cfDNA quantification.

[Response]

We appreciate the reviewer’s emphasis on the sub-percent range. While we cannot expand the dilution series immediately, we provide an analytical baseline using pre-transplant recipient samples (n=8), in which the true dd-cfDNA fraction is effectively zero. Using our donor-independent pipeline (without pre-transplant subtraction), the estimated dd-cfDNA averaged 0.06% (SD 0.02%). Treating these as blanks under CLSI EP17-A2, the preliminary limit of blank (LOB) is ~0.093% (mean + 1.645×SD). Hence, a 0.5% signal lies ~5× above the LOB and >20 SD above blank variability, supporting the analytical detectability of sub-percent changes without relying on clinical outcomes. We have added this baseline analysis to the Results and clarified in the Discussion that we will formally establish LOD/LOQ following CLSI EP17-A2 using replicated low-positive samples. In addition, we now include a brief theoretical sampling-error analysis (SE ≈ √{p(1−p)/(R·N_eff)}), showing that with our panel size and typical depth, the expected precision near 0.5% is consistent with our down-sampling data.

6. Use of Pre/Post-Transplant Difference Metric
The use of the differential allele ratio (post-transplant minus pre-transplant) for clustering is a central part of the method when baseline data are available. The authors assume this subtracts out background noise. However, this approach is highly sensitive to batch effects and technical variations between sequencing runs. The manuscript would be strengthened by providing quality control data demonstrating the technical reproducibility of the platform between runs, or by employing and validating an alternative normalization strategy

[Response]

We would like to clarify that the purpose of using the differential allele ratio (post-transplant minus pre-transplant) is not primarily to subtract background noise, but rather to define the fraction of dd-cfDNA as the net change relative to the recipient’s pre-transplant baseline. This formulation directly reflects the biological definition of donor-derived cfDNA, while also facilitating clustering of loci into reference-homozygous, heterozygous, and alternative-homozygous groups.

We acknowledge that this approach may also incidentally reduce certain sources of background variation. However, we do not agree that it is highly sensitive to batch effects or technical variation between sequencing runs. In our study, all samples were processed under uniform protocols and sequencing conditions, which minimizes potential run-to-run bias. Moreover, preliminary technical replicates in our dataset demonstrated high reproducibility (data not shown).

We agree that future work could be further strengthened by systematically evaluating technical reproducibility across sequencing batches, and by exploring alternative normalization strategies as an additional safeguard. We will include this point in the Discussion as a potential direction for future methodological improvements.

7. Substitute for Pre-Transplant Samples
The manuscript states: "If a pre-transplant recipient sample is not available, leukocyte-derived genomic DNA or DNA from other sources can be fragmentase-treated and used as a substitute." However, cfDNA and leukocyte-derived gDNA differ significantly in fragment length, structure, and distribution. How do the authors address the potential impact of these differences on the accuracy and reliability of the results?

[Response]

We would like to clarify that when pre-transplant cfDNA samples are unavailable, our protocol uses leukocyte-derived genomic DNA that has been enzymatically fragmented with fragmentase to approximate the size distribution of cfDNA (typically 150–180 bp). After this treatment, the DNA is double-stranded and within the expected cfDNA size range. Therefore, we do not anticipate significant structural differences that would affect PCR amplification or sequencing performance.

The reviewer’s concern regarding “distribution” is not entirely clear. If referring to fragment length distribution, this is effectively normalized by the fragmentase treatment. If referring to genomic origin biases, our SNP-targeted approach does not rely on cfDNA fragmentation patterns and is therefore less likely to be affected.

While we believe this strategy provides a practical substitute in cases where pre-transplant cfDNA cannot be obtained, we agree that direct comparisons between cfDNA and fragmentase-treated gDNA in future work would help further validate this assumption.

8. Sample Size and Statistical Power

Parent-Child Pairs: Given the limited sample size of only three parent-child pairs (n=3), conclusions drawn from this analysis should be treated with caution, and this limitation should be clearly stated in the Discussion.

Table 2: Lacks explicit indication of sample sizes, which should be added for clarity and transparency.

[Response]

We thank the reviewer for pointing this out. To improve clarity and transparency, we have revised the footnotes of Table 2 to explicitly state the sample sizes and relationship types represented (unrelated, sibling, and parent–child). Each category corresponds to a single representative case, and we have noted that for the parent–child pair only one example was available. Furthermore, because the number of time points with <10% dd-cfDNA was insufficient, CCC without baseline data could not be calculated in this group. This limitation has now been clearly acknowledged in the revised manuscript.

**9. Comparison with Existing Methods**

Although other methods are mentioned in the Introduction, it would be best to more directly compare this method with the gold standard in this field (such as ArcherDX’s LiquidIQ or Natera’s Prospera), highlighting its advantages and potential disadvantages in terms of process simplification, cost, and speed.
Moreover, the authors present their clustering method as a novel solution for dd-cfDNA quantification without donor genotype. However, the study fails to benchmark its performance against established donor-independent algorithms, such as:
Maximum likelihood estimation-based method using a binomial model [2]. A direct comparison on the same dataset(s) is essential to demonstrate the relative advantages, disadvantages, and potential improvements offered by the clustering approach in terms of accuracy, precision, and robustness, especially at low dd-cfDNA fractions

[Response]

We appreciate the reviewer’s suggestion to place our clustering approach in the context of existing donor-derived cfDNA assays. Direct comparisons with commercial platforms such as LiquidIQ (ArcherDX) or Prospera (Natera) were not possible in this study, as these assays are proprietary and not available in our setting. Nevertheless, conceptually our method differs in several respects: it does not require donor genotyping, which is a critical advantage in Japan where consent for donor DNA testing is often unobtainable, and it simplifies the computational process by employing clustering of allele ratios rather than mapping all reads to the entire genome. These features can reduce both turnaround time and analytical cost.

In terms of donor-independent estimation algorithms, a maximum likelihood estimation (MLE) approach based on a binomial model has been previously reported (PLoS Comput Biol, 2017). While MLE methods are powerful, they rely on specific distributional assumptions and may be more sensitive to noise in low-frequency alleles. In contrast, our clustering-based strategy is data-driven and leverages the natural separation of SNPs into homozygous and heterozygous groups. This provides a robust framework that performs well when sufficient numbers of informative SNPs are available. At very low dd-cfDNA fractions (<0.5%), both approaches share challenges, and direct benchmarking in future work will be valuable to further validate the relative advantages and limitations.

We have revised the Discussion to explicitly address these points and to highlight both the potential advantages and limitations of the clustering approach compared with existing assays.

**10. Graphical Enhancements**

It is recommended that trend lines or confidence intervals be added to the scatter plots in Figures 2, 3, and 4 to make the correlations more intuitive.

The time series diagram in Figure 5 suggests connecting the results of different methods with points of different shapes to more clearly observe the differences and consistencies among the three methods (direct method, clustering method 1, and clustering method 2). Additionally, Figure 5 lacks a clear legend to distinguish the different data series—adding one would significantly improve readability.

[Response]

We respectfully note that Figure 2(a–c) illustrates SNP-wise allele fraction distributions and is not suitable for regression lines. To address the reviewer’s request, we have instead added regression lines with 95% confidence intervals to the correlation plots of expected versus estimated dd-cfDNA fractions. These are provided as new Supplementary Figures S1 Fig. – S3 Fig., which clearly show the linear relationship and precision of the clustering-based estimates. And we re-designed Figure 5 with shape-coded series and a clear legend for immediate visual comparison.
