# Supplementary material for: A SNP-based capture and clustering workflow to assess donor-derived cell-free DNA in transplantation
Source: PLoS One. 2026 Feb 2;21(2):e0342082. doi: 10.1371/journal.pone.0342082 (PMC12863547; doi:10.1371/journal.pone.0342082)
Supplement: Attachment — Submitted filename: Response to Reviewer 2_YY.docx [file pone.0342082.s005.docx]

**Reviewer 2:**

We sincerely thank Reviewer 2 for your constructive comments and insightful suggestions. We have revised the manuscript accordingly to improve clarity, rigor, and clinical relevance. Below, we provide a detailed point-by-point response.

**1. Clinical Correlation and Utility**

I appreciate that the authors added a clinical case (S4 Fig) illustrating dd-cfDNA changes. However, in this figure, the orange line (w/o pre-data) lacks data points where dd-cfDNA levels exceed 10%, especially around the event biopsy time point. Since rejection is a dynamic process, missing data at these clinically relevant points limits interpretation. The authors are encouraged to supplement these data if available.

In addition, the figure currently lacks axis labels for both x- and y-axes. Each figure should be self-explanatory so that readers can understand its meaning even without referring to the caption.

Finally, while I understand that a direct comparison with existing dd-cfDNA assays was beyond the current scope, such a comparison would be highly valuable for future work, as it would strengthen the clinical relevance and translational potential of this new method.

[Response]

We thank the reviewer for the insightful comments.

(1) Missing high dd-cfDNA data points:

We acknowledge that the orange “clustering_wo pre-data” line in Supplementary Figure S4 lacks values above 10%. As discussed in the manuscript, when pre-transplant reference data are unavailable, dd-cfDNA estimates ≥10% become unreliable due to reduced cluster separability. For this reason, values in that range were intentionally excluded from the plot. To improve transparency, we have added a clarification to the figure legend and provided the two relevant estimates as reference values. Specifically, the w/o pre-data estimates at day 176 and day 252 were 8.55% and 10.79%, respectively; however, because values approaching or exceeding 10% are prone to reduced reliability in the absence of pre-transplant data, these points remain omitted from the plotted curve. This approach preserves interpretability while avoiding the visual presentation of estimates that may not accurately reflect the true biological signal.

Revised figure legend:

Time-series plot of dd-cfDNA (%) in a representative liver transplant recipient. The blue line represents dd-cfDNA estimates obtained using the clustering-based method with pre-transplant baseline data, and the orange line represents estimates obtained without pre-transplant data. A rejection episode confirmed by biopsy occurred at the point indicated by the downward arrow (“event biopsy”). During the rejection period, dd-cfDNA levels showed a transient increase. Below 10%, estimated values were nearly identical regardless of whether pre-transplant reference data were used. As noted in the Discussion, dd-cfDNA estimates ≥10% obtained without pre-transplant reference data were excluded from the plot because reduced cluster separability leads to decreased reliability in this range. For reference, the clustering-based estimates without pre-transplant data at day 176 and day 252 were 8.55% and 10.79%, respectively; these values are provided in the legend for transparency but are not shown in the figure due to their limited reliability.

(2) Figure labeling:

We have added x-axis (“Days after transplantation”) and y-axis (“dd-cfDNA fraction (%)”) labels to ensure that the figure is self-explanatory.

(3) Future comparison with commercial assays:

We fully agree that direct comparison with existing commercial dd-cfDNA assays would strengthen the translational relevance of this work. A statement has been added in the Discussion highlighting this as an important next step for future validation studies.

Added sentence in Discussion:

Importantly, while direct comparison with existing commercial dd-cfDNA assays was beyond the scope of this study, such cross-platform evaluation will be a key future step to benchmark analytical concordance, turnaround time, and cost efficiency. Future studies with larger and more diverse patient cohorts will further validate the generalizability and clinical utility of this clustering-based method.

**2. Cost and Efficiency**

I would like to update the authors’ understanding regarding existing methods: commercially available dd-cfDNA assays that do not require donor genotyping are already in widespread use.

In the Discussion, the authors briefly mention relative cost and efficiency advantages, but I could not find detailed information about cost aspects in the revised manuscript. Since cost and reliability are key determinants for clinical adoption, a short discussion on the economic feasibility or expected cost differences compared to existing commercial assays would be appreciated.

[Response]

We thank the reviewer for the important comment.

We have revised the Discussion to clarify that multiple donor-independent dd-cfDNA assays (e.g., AlloSure, Prospera) are already commercially available and widely used in clinical practice.

We also added a short paragraph discussing the relative economic feasibility of our method. Because no formal cost-analysis study was performed in the present work, we avoided providing specific numerical estimates. Instead, we now state that our workflow is designed to minimize reagent use and analytical overhead by eliminating genome-wide mapping and donor genotyping steps. Furthermore, it can be implemented on standard benchtop NGS platforms without requiring proprietary software pipelines. We believe these features have the potential to reduce overall operational burden, although a direct cost comparison with existing commercial assays will require a future dedicated evaluation.

**5. Low-Level Detection and Accuracy**

While I understand the authors’ rationale, the potential measurement error in the 0–1% dd-cfDNA range remains clinically concerning. For example, in lung transplantation, a 1% threshold is often used to indicate possible rejection. In Table 1, when the actual mixing rate is 0.5%, the measured value is 1.0%, which could lead to significant clinical misinterpretation. This limitation should be clearly discussed, as small inaccuracies around this threshold can greatly influence clinical decision-making.

[Response]

We thank the reviewer for raising this important point regarding low-level dd-cfDNA measurement accuracy.

We fully agree that small deviations in the 0–1% range may have clinical implications, particularly in organ types such as lung transplantation where a threshold of ~1% is commonly used to indicate possible rejection. In our spike-in experiment, the estimated value for the 0.5% mixture was indeed slightly overestimated at 1.0%. We acknowledge that such variation could influence interpretation in clinical settings that rely on rigid thresholding.

To address this concern, we have added a clear discussion in the revised manuscript. Specifically, we now note that:

- small absolute deviations (e.g., ±0.3–0.6%) can produce proportionally large relative changes in the sub-1% range;
- this effect is not unique to our clustering-based method and is also observed in other dd-cfDNA assays due to assay noise, sampling variance at very low allele frequencies, and Poisson limitations inherent to cfDNA fragment counts;
- therefore, absolute thresholds near 1% should be interpreted with clinical context in mind, and trends over time may provide more reliable information than single-point measurements in this range.

Importantly, despite the small overestimation at 0.5%, all clinically relevant increases (e.g., >2–3%) in both the spike-in dataset and clinical cohort showed strong concordance with rejection status. We now highlight that while our method performs robustly across the clinically relevant spectrum, caution is warranted when interpreting values extremely close to 1%, and this represents an important area for future methodological refinement.

A corresponding paragraph has been added to the Discussion to explicitly acknowledge and contextualize this limitation.

**9. Comparison with Existing Methods**

Our earlier comment was intended to encourage comparison with existing commercial dd-cfDNA platforms to highlight the advantages and limitations of the new method. Although the authors indicated that direct comparison was difficult, I still suggest that, if feasible in future work, they test a subset of samples using a commercial platform to compare turnaround time and measurement concordance. This would significantly strengthen the paper’s translational impact.

[Response]

We appreciate the reviewer’s constructive suggestion. We have added a statement in the Discussion noting that future studies will include direct comparisons with commercial platforms (e.g., AlloSure, Prospera) to evaluate concordance and turnaround time using the same clinical samples. We believe this will provide a robust benchmark for translational validation once access to such assays is available.

**10. Graphical Enhancements**

It is recommended that trend lines or confidence intervals be added to the scatter plots in Figures 2, 3, and 4 to make the correlations more intuitive.

The time series diagram in Figure 5 suggests connecting the results of different methods with points of different shapes to more clearly observe the differences and consistencies among the three methods (direct method, clustering method 1, and clustering method 2). Additionally, Figure 5 lacks a clear legend to distinguish the different data series—adding one would significantly improve readability.

[Response]

We thank the reviewer for this constructive suggestion.

Among the figures in the main manuscript, the only panels that present correlation scatter plots are Figure 4(a–c). Following the reviewer’s recommendation, we have generated three new supplementary figures (S1–S3) in which we added linear regression lines and 95% confidence intervals to the corresponding correlation plots:

- **S1 Fig.**: Full-range (0–100%) expected vs. estimated dd-cfDNA (pre-transplant data used)
- **S2 Fig.**: 0–10% clinically relevant range (pre-transplant data used)
- **S3 Fig.**: 0–10% expected vs. estimated dd-cfDNA (no pre-transplant data)

These supplementary figures provide a clearer visualization of the linearity, accuracy, and variance of the estimation.

The main Figure 4 remains unchanged, as the reviewer’s request is now fully addressed in the Supplementary Figures.
