# Supplementary material for: A SNP-based capture and clustering workflow to assess donor-derived cell-free DNA in transplantation
Source: PLoS One. 2026 Feb 2;21(2):e0342082. doi: 10.1371/journal.pone.0342082 (PMC12863547; doi:10.1371/journal.pone.0342082)
Supplement: Attachment — Submitted filename: Response to Reviewer 12192025.docx [file pone.0342082.s006.docx]

**Response to Reviewer #2**

We sincerely thank Reviewer #2 for the careful re-evaluation of our revised manuscript and for the constructive and insightful comments. We believe that addressing these points has substantially improved the clarity, clinical relevance, and translational positioning of our work. Our detailed responses are provided below.

**1. Clinical Correlation and Utility**

**Reviewer comment:**

The orange line (w/o pre-data) in Supplementary Figure S4 lacks data points exceeding 10%, particularly around the biopsy-proven rejection event. In addition, axis labels are missing. A comparison with existing assays would also strengthen clinical relevance.

**Response:**

We thank the reviewer for these important observations.

First, we have revised Supplementary Figure S4 to include explicit axis labels for both axes (x-axis: *Time relative to biopsy (days)*; y-axis: *dd-cfDNA (%)*), ensuring that the figure is fully self-explanatory without reference to the caption.

Regarding the absence of >10% dd-cfDNA data points in the *w/o pre-data* (orange) curve, we would like to clarify that these values were not missing from the dataset but were intentionally not displayed in the plotted time series due to reduced estimation reliability in this range. Specifically, when pre-transplant reference data are unavailable, higher dd-cfDNA fractions may reduce cluster separability in the recipient-only clustering framework, leading to less robust estimation. To avoid over-interpretation of potentially unstable values, these estimates were omitted from the plotted curve and instead reported in the figure legend for transparency.

To maintain transparency, we now report the corresponding *w/o pre-data* estimates in the legend (e.g., 8.55% at day 176 and 10.79% at day 252), while explaining why values in this range are not displayed in the figure. We also emphasize in the Discussion that future prospective studies—including denser longitudinal sampling and, where feasible, cross-platform comparisons with established commercial dd-cfDNA assays—will be important to further validate performance across the full dynamic range.

**2. Cost and Efficiency**

**Reviewer comment:**
Donor-independent commercial dd-cfDNA assays are already in widespread use, and the revised manuscript lacks sufficient discussion of cost considerations.

**Response:**

We appreciate the reviewer’s clarification and apologize for any ambiguity in our original wording. We have revised the manuscript to clearly acknowledge that donor-independent commercial dd-cfDNA assays are already widely available and clinically implemented.

Although we did not perform a formal cost analysis, we have expanded the Discussion to provide a methodological perspective on economic feasibility. Specifically, we now explain that our workflow is based on a targeted SNP capture panel (~300 loci) and simple clustering-based analysis, eliminating donor genotyping and genome-wide sequencing. This design reduces sequencing depth requirements and allows implementation on standard benchtop NGS platforms using open-source software, in contrast to commercial assays that rely on proprietary algorithms and specialized infrastructures.

We emphasize that these considerations suggest potential advantages in operational simplicity and scalability, while refraining from quantitative cost claims. A formal head-to-head cost and efficiency comparison with commercial platforms is identified as an important objective for future work.

**3. Population Specificity and Generalizability**

**Reviewer comment:**
Thank you for your clarification.

**Response:**

We thank the reviewer for the acknowledgment and have no further changes to add.

**4. SNP Panel Design and Sequencing Parameters**

**Reviewer comment:**

Thank you for your clarification.

**Response:**

We appreciate the reviewer’s confirmation and have no additional modifications regarding this point.

**5. Low-Level Detection and Accuracy**

**Reviewer comment:**

Measurement error in the 0–1% dd-cfDNA range may lead to clinically significant misinterpretation, particularly around commonly used thresholds such as 1%.

**Response:**

We thank the reviewer for highlighting this clinically important issue and fully agree that uncertainty in the low dd-cfDNA range warrants careful discussion.

In response, we have added a dedicated paragraph to the Discussion explicitly addressing measurement variability in the 0–1% dd-cfDNA range. As noted by the reviewer, in Table 1 a nominal mixing ratio of 0.5% resulted in an estimated dd-cfDNA value of approximately 1.0%. We clarify that this variability reflects stochastic variation in read sampling and cluster assignment that becomes more pronounced at very low allele fractions.

Importantly, we now emphasize that dd-cfDNA values close to commonly used clinical decision thresholds should not be interpreted in isolation, but rather evaluated in conjunction with longitudinal trends and the overall clinical context. We note that such considerations are particularly relevant in transplant settings where clinical decision thresholds around this range are commonly applied. We further emphasize that the primary strength of the proposed clustering-based framework lies in capturing relative temporal changes within individual patients, rather than providing absolute precision at the lowest detectable range.

Finally, we note that future methodological refinements, increased sequencing depth, and prospective validation studies will be required to further improve robustness and accuracy in this clinically sensitive low-level range.

**6–8. Use of Pre/Post-Transplant Difference Metric, Substitute for Pre-Transplant Samples, Sample Size and Statistical Power**

**Reviewer comment:**

Thank you for your responses.

**Response:**

We appreciate the reviewer’s acknowledgment and have no further changes to add.

**9. Comparison with Existing Methods**

**Reviewer comment:**

A future comparison with commercial dd-cfDNA platforms would significantly strengthen translational impact.

**Response:**

We fully agree with the reviewer. While direct comparison with commercial platforms was beyond the scope of the current study, we have now explicitly addressed this point in the Discussion. Specifically, we acknowledge the widespread clinical use of donor-independent commercial dd-cfDNA assays and clarify that our clustering-based approach is intended as a complementary and transparent workflow rather than a direct replacement. We further state that future prospective studies evaluating a subset of samples using both our method and established commercial assays will be essential to assess analytical concordance, turnaround time, and appropriate clinical use cases.

**10. Graphical Enhancements**

**Reviewer comment:**

Thank you for your response.

**Response:**

We thank the reviewer for the positive feedback and confirm that the requested graphical clarifications have been implemented.
